# Impact of Mouthwash Immersion on 3D-Printed Resin Material: A Comprehensive Evaluation of Surface Hardness, Surface Roughness, Wettability, and Characterization Using FTIR and FE-SEM Techniques

**DOI:** 10.4317/jced.63496

**Published:** 2026-01-28

**Authors:** Esraa Fadhil Abass, Abdalbseet A. Fatalla, Matheel AL-Rawas, Johari Yap Abdullah

**Affiliations:** 1 Department of Prosthodontics, College of Dentistry, University of Baghdad, Baghdad, Iraq; 2 Prosthodontic Unit, School of Dental Sciences, Universiti Sains Malaysia, Kubang Kerian, Malaysia; 3 Craniofacial Imaging Laboratory, School of Dental Sciences, Health Campus, Universiti Sains Malaysia, Kubang Kerian, Kota Bharu 16150, Malaysia; 4 Dental Research Unit, Center for Transdisciplinary Research (CFTR), Saveetha Dental College, Saveetha Institute of Medical and Technical Sciences, Saveetha University, Chennai 602105, India

## Abstract

**Background:**

This study examined how mouthwashes affect the surface hardness, roughness, and wettability of 3D-printed resin material FreePrint® 3D Printing Material (Dental Med Resin Crown).

**Material and Methods:**

40 samples were divided into four groups for each test. The samples were stored in distilled water (control) and Kin Forte, Lacalut Active, and Corsodyl mouthwashes for three months. Every sample was controlled by a robotic arm during immersion. To simulate daily usage, each sample was immersed twice a day for one minute. After immersion, sample hardness, roughness, and wettability were assessed. There were FTIR and FE-SEM tests performed for characterization purposes.

**Results:**

Mouthwashes increased surface roughness but did not affect surface hardness. In intergroup comparisons, Kin and Lacalut exhibited a rougher surface than Corsodyl. Hardness assessment groups were not significantly different. One-way ANOVA supported these findings, showing that roughness differences were significant (p=0.01) while hardness differences were not (p=0.879).

**Conclusions:**

The mouthwashes reduced the 3D-printed material's surface roughness but not its hardness. Some mouthwashes may change the surface properties of 3D-printed resins, potentially diminishing positive outcomes in the context of prosthodontics.

## Introduction

Computer-aided design and manufacturing (CAD-CAM) technology has a huge influence on restorative dentistry and continues to develop with subtractive and additive manufacturing approaches. Subtractive manufacturing (SM) of a solid block wastes material and wears tools, making it problematic. Additive manufacturing (AM), or 3D printing technology, creates a solid 3D item layer by layer, allowing for quick, wasteless prosthesis fabrication. So it's no surprise that dentistry embraces this technology (1). Dentistry widely utilizes stereolithography (SLA) and digital light projection (DLP) (2 , 3). 3D-printed resins have better mechanical qualities and reduced fungal adherence than conventional materials, making them appropriate for clinical applications (4). Mechanical cleaning and mouthwashes reduce plaque, cavities, and gingivitis, particularly in hard-to-reach areas (5). Mouthwashes affect dental restorations differently depending on their composition. Various mouthwashes increase the surface roughness of the dental restorations (6 , 7). According to the printing technique employed, including post-curing and resin chemical composition, mechanical qualities are affected by these factors (8). A wide variety of mouthwashes exists on the market; nevertheless, many of them have not been examined for their impact on resin materials. Moreover, there is an absence of research explicitly contrasting the effects of Kin, Lacalut, and Corsodyl on 3D-printed resin. The objective of this research is to evaluate changes in surface roughness, surface hardness, and wettability, alongside characterization by FTIR and FE-SEM methods, to analyze and compare the effects of immersing 3D-printed resin materials in Kin, Lacalut, and Corsodyl commercial mouthwashes. The hypothesis to be tested is that the physical characteristics of 3D-printed resin are unaffected by immersion in various mouthwashes.

## Material and Methods

1. Sample size calculation and sample preparation The sample size calculation was determined using G*power 3.1.9.6 at a 0.05 significance level (), 0.6 effect size, 0.8 power, and four groups per test. The total sample size was 36, with 9 samples per group per test. The final sample size consisted of ten samples per immersion group for each test, totaling 40 samples per test. For the microhardness test, 40 bar-shaped samples (65 × 10 × 2.5 mm) were fabricated according to standardized dimensions, while an additional 40 samples (65 × 10 × 2.5 mm) were designated for the roughness test. Furthermore, 40 disc-shaped samples (15 mm in diameter and 3 mm in thickness) were prepared for the contact angle measurement, according to ISO 4049:2019 specifications. The 3D printed samples were fabricated using FreePrint® 3D Printing Material (Dental Med Resin Crown) (A3 shade). To guarantee the resin's homogeneity, it was first processed in an LC-3D mixer for 15 minutes. The resin was then transferred to a DLP-based 3D printer (ASIGA Max UV-385, Australia) for the printing process. The samples were processed in the 3D printing software using the STL format (Asiga Composer). Each layer was printed with a thickness of 50 µm at an angle of 90°, a light intensity of 6.1 mW/cm², and a duration of 2.15 s. All the 3D printing procedures were conducted in accordance with the manufacturer's guidelines. After completing the printing process, all samples were immersed in isopropanol (99.9%) for 10 minutes in a washing device (Ultrasonic cleaner, China). Subsequently, the samples were coated with pure glycerin solution and allowed to dry for a while. The samples were then placed in a glycerin container for post-curing in an Otoflash G171 system. The procedure was conducted with two 2000-flash cycles in an inert atmosphere of nitrogen gas, with the component turning after each cycle. After the post-curing step, the additional supporting structures around the samples were removed using a low-speed rotary instrument. Then, the samples in a wet condition were polished using a polishing machine. Surface finishing was carried out at this stage to remove excessive roughness and imperfections introduced to the samples' surfaces during manufacture. Each individual specimen was visually examined, and those samples with surface defects, warping, or surface irregularities were excluded to ensure consistency and accuracy of the results. 2. Immersion process This research examined three different types of mouthwash: Kin Forte, Corsodyl, and Lacalut Aktiv. Additional information can be found in Table 1.

Table 1

This research included robotic arm immersion equipment to simulate mouthwash application. This device was used to guarantee that immersion conditions and mouthwash exposure accurately reflected real mouthwash usage. Each sample was immersed 180 times, twice daily for one minute, approximating 90 days of mouthwash exposure. A total of ten samples of each mouthwash were used for the immersion stage. For each mouthwash, immersion was carried out using two 500 mL containers, each containing 300 mL of liquid. One bottle contained mouthwash, while the other contained distilled water. Following the immersion in the mouthwash container, the samples were washed with distilled water to eliminate any residual mouthwash. After completing all the immersion cycles, the samples underwent a variety of tests to assess the mouthwash's impact on the surface properties and characteristics of the 3D-printed resin. The mouthwash solution was changed every four hours to prevent the loss of its active components. This occurs because, after prolonged periods of using the mouthwash, some substances may evaporate or other forms of contamination may arise. 3. Characterization tests 3.1 Fourier Transform Infrared Spectroscopy (FTIR) FTIR is an effective method for identifying specific functional groups within a compound and for elucidating its structural characteristics in greater detail. This method identifies specific wavelengths of infrared light that are absorbed, allowing for a study of potential chemical interactions between different mouthwash solutions and the polymer matrix. The specimens were first coated with KBr (potassium bromide) and positioned in the sample holder for scanning within the range of 4000 to 400 cm-¹ wave numbers, from which their FTIR spectra may be determined. Four specimens were produced and evaluated from each group (n = 4). 3.2 Field-emission scanning electron microscopy (FE-SEM) The samples were prepared for SEM examination, with three samples designated for each group, and were examined post-immersion. In compliance with the relevant technical specifications, the square samples were sectioned into 3 × 3 mm squares and coated with 40-60 nm of gold. A microscope was used to examine the sample at 2000X and 4000X magnifications, with an acceleration voltage of 30 kV. 4. Experimental tests 4.1 Surface roughness test A stylus-type electronic roughness tester (JITA 810, China) was used to evaluate surface roughness in compliance with ISO 4287. The device used a diamond-tipped stylus with a 5 m tip radius. The stylus traversed a total distance of 4.0 mm at a velocity of 0.5 m/s, exerting a measuring force of 4 N. A cut-off value of 2.5 mm was chosen for surface roughness. The mean value was calculated from five measurements. 4.2 Surface hardness test The indentation hardnesses were assessed using a Shore D durometer (HLX-AC, China), in accordance with ASTM D2240. A force of 44.5 N was applied using a conical indenter with a hemispherical tip with a radius of 0.8 mm. The indenter was connected to a digital scale to measure the hardness. Each sample was indented five times at different positions, and the average value was calculated. 4.3 Wettability test The contact angle was measured using the sessile drop method. A contact angle goniometer (Model CAM110P, Creating Nano Tech, Si-plasma) was used. A tiny droplet of distilled water, measuring between 3 and 10 µL, was applied to the horizontally oriented sample surface. Fifteen seconds later, a picture of the droplet was obtained, and the contact angle was then determined using specialized image processing software that automatically formed the tangent line at the three-phase boundary (solid-liquid-air) to ascertain the contact angle. The contact angle was measured three times at various locations on each sample, and the average value was recorded. 5. Statistical analysis Data analysis was carried out using SPSS software, version 23 (IBM Corp., Chicago, USA). The Shapiro-Wilk test evaluated the normal distribution, whereas Levene's test determined the appropriate post hoc test. One-way ANOVA and Tukey's post hoc analysis were used. A score of 0.05 was set as the point of statistical significance.

## Results

**Figure 1:**
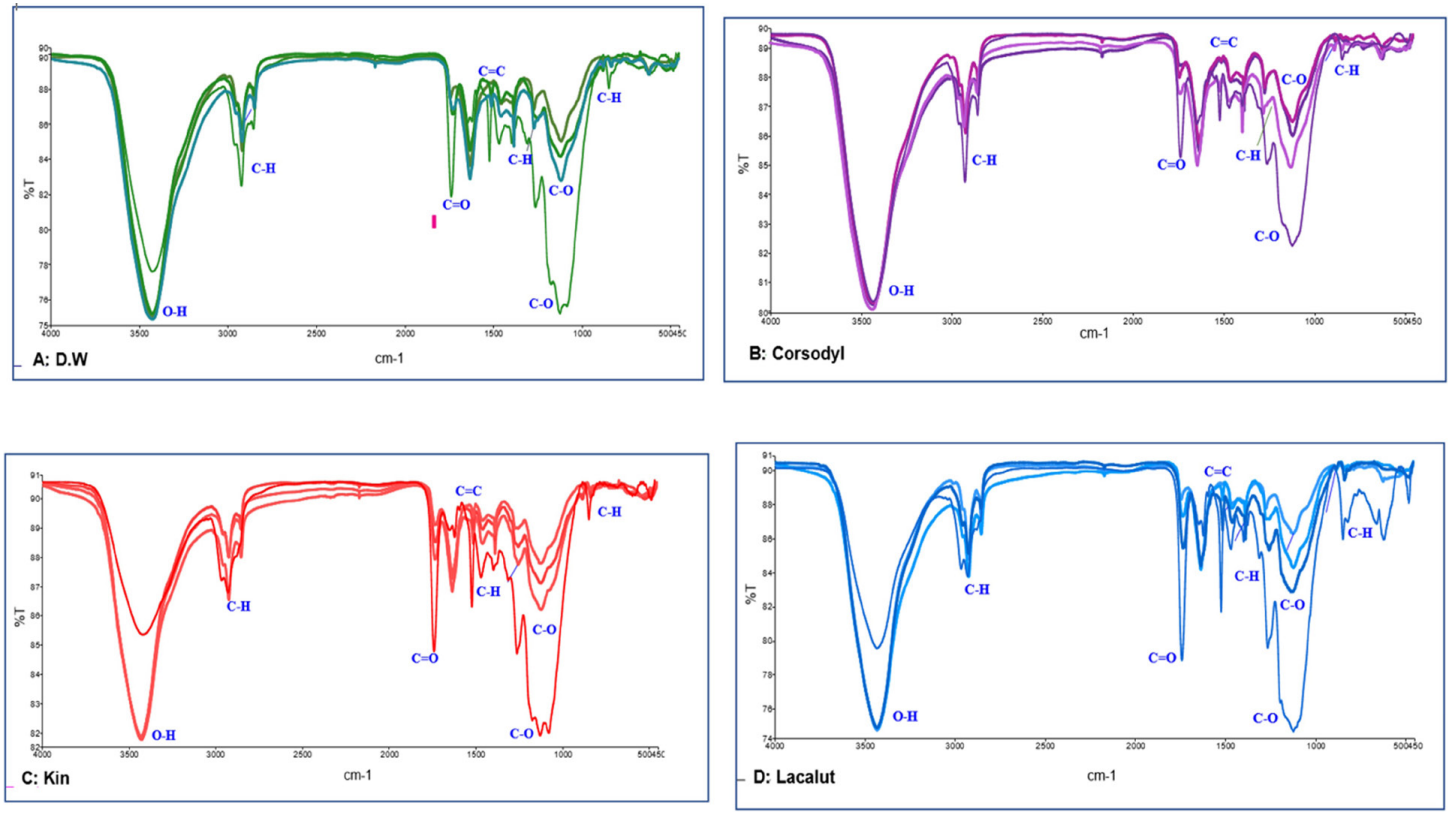
A. FTIR of DW Samples. B. FTIR of Corsodyl Samples. C. FTIR of Kin Samples. D. FTIR of Lacalut Samples.

1. FTIR results The FTIR spectra (Fig. 1) demonstrate that immersion in various mouthwashes did not alter the spectral range of the 3D-printed resin, indicating the absence of any chemical reaction.

1

**Figure 2:**
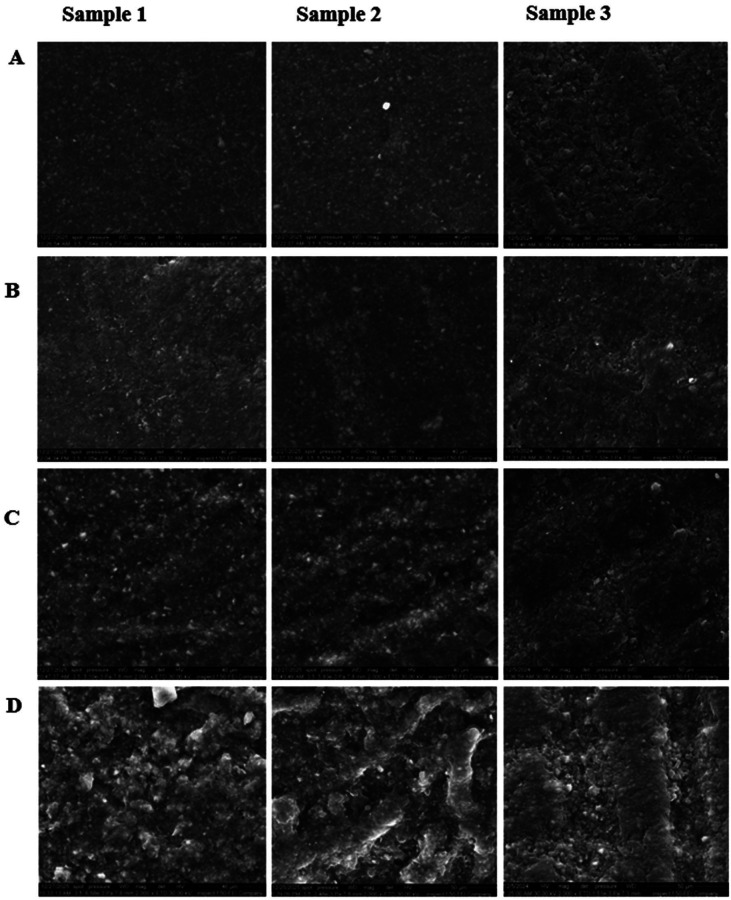
The surface of 3D-printed resin in typical SEM images, at 2000x magnification. A: Control sample immersed in DW, B: sample immersed in Corsodyl mouthwash, C: sample immersed in Kin mouthwash, D: sample immersed in Lacalut mouthwash.

**Figure 3:**
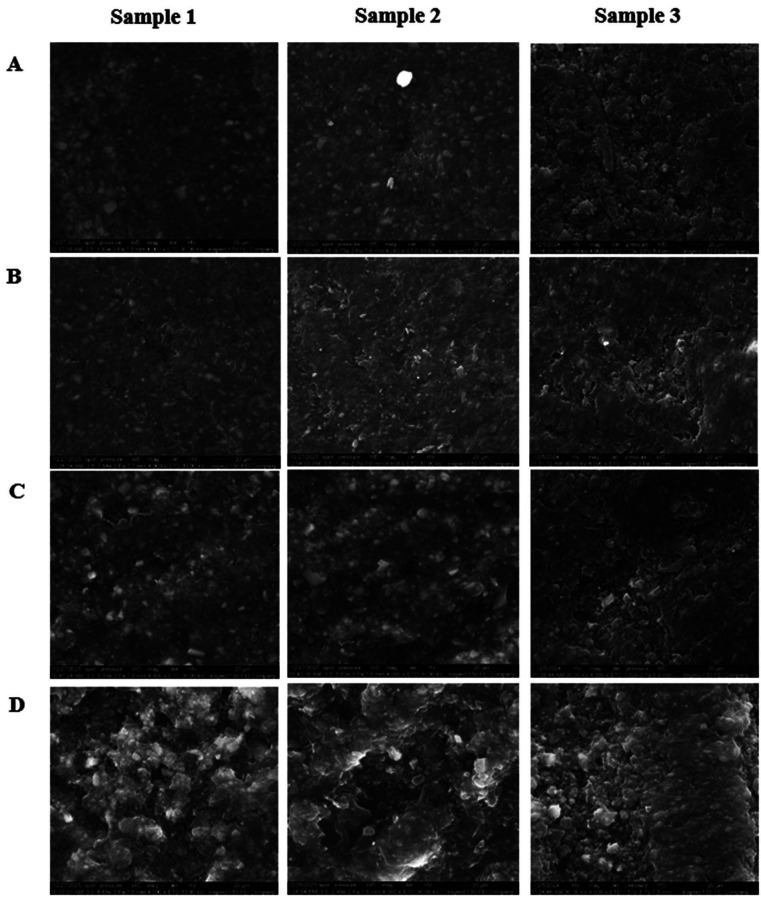
The surface of 3D-printed resin in typical SEM images, at 4000x magnification. A: Control sample immersed in DW, B: sample immersed in Corsodyl mouthwash, C: sample immersed in Kin mouthwash, D: sample immersed in Lacalut mouthwash.

The FTIR was used to ascertain the existence of functional groups and to assess potential alterations in the polymer matrix, particularly to determine whether any chemical interactions occurred. The spectra exhibited distinctive absorption peaks associated with functional groups in the samples. A signal between 3420 and 3436 cm-¹ was seen in the majority of samples, ascribed to O-H stretching vibrations of hydroxyl groups. Peaks at 2922-2936 cm-¹ and 2854-2855 cm-¹ were attributed to C-H stretching vibrations. A peak at 1727-1744 cm-¹ corresponds to the C=O stretching vibrations of ester functional groups. Peaks at around 1610-1649 cm-¹ were seen in some samples, attributed to C=C stretching vibrations of alkene (carbon-carbon double bond) groups. The peaks appeared at 1511, 1455-1460 cm-¹, 1384 cm-¹, 1107-1298 cm-¹, 720-879 cm-¹, and 400-600 cm-¹, corresponding to C=C, -CH2/-CH3, -CH3, C-O-C, vinyl/methylene C-H, and fingerprint vibrations, respectively. Minor discrepancies in peak appearance within the constrained range across samples may result from experimental factors, including spectral resolution or noise during FTIR data collection. Furthermore, owing to residual monomer content or minor variations in polymerization, rather than chemical alterations. No new peaks emerged, no existing peaks vanished, and peak locations remained stable. The interaction between the mouthwash and the resin matrix was restricted to physical interactions, including van der Waals forces and hydrogen bonding, demonstrated through small changes in the intensity of absorption bands and tiny displacements in the vibrations of existing connections. 2. SEM results The SEM images of the samples immersed in distilled water exhibited no indications of deterioration, as shown in Fig. 2, Fig. 3A.

2

3

Samples immersed in Corsodyl had comparable surface topographies to the DW group, with a slightly roughened surface as seen in Figs. 2, 3B. On the other hand, the samples immersed with Kin mouthwash exhibited greater surface roughness and minor surface imperfections can be observed in Fig. 2, Fig. 3C. The sample immersed in Lacalut exhibited more significant surface alterations, marked by increased roughness and more prominent surface pits, as shown in Figs. 2, 3D. The observed discrepancies in SEM surface morphology among samples within the same group can primarily be ascribed to the intrinsic properties of the 3D printing process, which involve layer-by-layer fabrication and localized variations in polymerization, leading to inherent surface heterogeneity. Despite the standardization of finishing and polishing techniques for all samples, some localized variations in surface reaction may nevertheless arise. Moreover, SEM-related imaging artifacts may affect the surface appearance without reflecting true changes in the material structure. 3. Roughness test results The mean Ra values for the samples are shown in Table 2.

Table 2

A one-way analysis of variance revealed a significant impact of the immersion solutions on surface roughness (F = 6.389, p = 0.001). Subsequent to the findings of Levene's test (p &gt; 0.05), statistically significant differences were seen only in the pairwise comparisons between the DW group and both the Kin and Lacalut groups, as well as between Corsodyl and Lacalut (p &lt; 0.05). The other comparisons lacked statistical significance (p &gt; 0.05). 4. Hardness test results Table 3 presents the mean values of surface hardness.

Table 3

A one-way ANOVA was performed, revealing no statistically significant differences among the groups (F = 0.224, p = 0.879), indicating that exposure to various mouthwashes did not substantially impact the hardness of the examined resin. No pairwise comparisons among groups were conducted. 5. Wettability test results Table 4 presents findings among the evaluated groups.

Table 4

A one-way ANOVA demonstrated a substantial impact of the immersion solutions on wettability (F = 87.61, p = 0.000). The post-hoc analysis (Tukey's HSD test) indicated a statistically significant difference among the majority of the groups (p &lt; 0.05), with the exception of the comparison between Corsodyl and Kin (p &gt; 0.05).

## Discussion

Evidence-based data showed that the deposition of plaque was markedly reduced when chlorhexidine-containing mouthrinses were used alongside standard toothbrushing and cleansing for durations of 4 to 6 weeks or 6 months. No data indicated that any concentration or intensity of chlorhexidine rinse was more effective than another (9). However, for dental appliances and restorations, different solutions considerably affected microhardness and surface roughness (10 , 11). Surface roughness is a critical feature in material assessment, since it may facilitate plaque formation (2 , 12). This research indicated that all 3D printed materials exhibited a general increase in surface roughness (Ra). Previous research indicated that surface-finishing procedures such as disks, polishing kits, and polishing pastes significantly influence the surface roughness of almost all restorative materials (13). The study indicated that surface roughness is markedly enhanced by glazing and the use of surface-sealing agents, which were not utilized in this research (13). Previous studies have shown that 3D-printed resins have superior Ra values when compared with conventional resin and CAD-CAM milled resin (14). Despite the increased surface roughness in the 3D-printed samples, further research indicated that 3D-printed resins may be more suitable for extended clinical use and provide superior treatment results for patients (8). The mean Ra values of the samples immersed in the three mouthwashes indicated that these solutions significantly altered the surface roughness of the 3D-printing resin material used. Although the alterations were imperceptible to the naked eye, the increase in surface roughness was apparent in the SEM pictures when compared with the smoother look of the control samples. These microscopic changes may enhance plaque retention or staining; however, in vivo validation is necessary (15 , 16). A notable factor affecting the resin may be the chemical compositions of the mouthwashes (15). The present study demonstrated that immersion influenced the 3D-printed resin, irrespective of the type of mouthwash solution used. This is mostly attributed to mouthwash with low pH and the hygroscopic properties of resin-based products. This altered the top layer of resin, revealing a subsurface area and resulting in a coarser texture. This aligns with a prior in vitro investigation by Golfeshan et al., which assessed the impact of herbal and chlorhexidine mouthwashes on the surface roughness of orthodontic acrylic resin, concluding that mouthwashes with the lowest pH caused the most significant increase in surface roughness (17). This research found that 3D-printed resin material exhibited no significant variation in microhardness following immersion in mouth rinses. Despite the acidic properties of mouthwashes, no significant changes were seen, perhaps because the acidity was insufficient to impact the mechanical properties of the resin materials. This contrasts with Hazar et al.'s in vitro investigation on CAD/CAM composite materials, which indicated a reduction in microhardness after immersion in antiviral mouthwashes. The variations, attributed to material composition, mouthwash formulation, and exposure conditions, did not substantially impair the mechanical capabilities of the 3D-printed resin in the evaluated mouthwashes (18). Corsodyl was the only mouthwash used that has a low alcohol concentration of 7.6% ethanol. Alcohol in mouthwash may lead to the degradation of resin components. Ethanol will enhance hydrolytic breakdown by softening the matrix resin, resulting in a plasticizing impact that expands the polymer chains and increases plastic deformation. This validates the previously mentioned softening and plasticization impact of ethanol on acrylic resins. The findings of this investigation indicated no substantial impact on the microhardness of the 3D-printed resin attributable to the low ethanol content and brief exposure period (19 , 20). The examined resin demonstrates intrinsic high hardness, in contrast to conventional resin, which may have enhanced its resistance to alterations caused by mouthwash exposure. Furthermore, the period of the present study (intermittent immersion for three months) was a relatively short comparison to previous research (continuous immersion for six months to one or even two years). The duration of immersion may have contributed to the change in hardness noted by George et al. The immersion of esthetic restorative material in an in vitro investigation for a continuous 24-hour duration influences the material's hardness (19 , 21). Extended intermittent immersion could reveal additional intriguing information. Wettability is an essential factor in assessing the wetting behavior of solid surfaces by evaluating the contact angle of a liquid on the surface in consideration. The 3D-printed resin material examined in this work exhibited a contact angle () of 82.14°, signifying a hydrophilic surface ( &lt; 90°) (24). The evaluated mouthwashes demonstrated enhanced hydrophilicity by decreasing the contact angle. Modifications to the chemical and physical properties of polymeric materials, such as molecular weight, polydispersity, crystallinity, thermal transitions, and thermal degradation, may lead to variations in wettability. The characteristics and matrix polymer shape are directly correlated with the wettability and surface free energy discussed in (25). The contact angle may be influenced by the printing method, including the orientation of the lamination and the thickness of the layers. Reports indicated that vertically laminated surfaces exhibit much higher contact angles compared to horizontally laminated surfaces and that an increase in layer thickness correlates with a reduction in liquid spreading, as reported by Kang et al. The samples used in this investigation were printed at a 90° orientation with a layer thickness of 50 µm (26). The findings of the present research agree with those of Pogorzelski et al., who observed that immersing PMMA in commercial mouthwashes resulted in a reduction of contact angle. The surface roughness of the 3D-printed resin in this investigation increased upon immersion, possibly contributing to the observed decrease in contact angle. This reduction may result from many processes, including the adherence of liquid constituents, irreversible adsorption inside solid pores, chemical erosion of polymer surfaces, etching and the formation of micro-roughness, and the leaching of more soluble PMMA components (24). As surface roughness intensified, the material exhibited enhanced hydrophilicity, resulting in a reduced contact angle. As a result, the surface energy and wettability enhanced, facilitating improved fluid dispersion and homogeneity over the surface (27 - 29). This research has certain limitations. Clinically, mouthwash can have different effects on 3D-printed materials depending on things like nutrition, dental care products, biofilm formation, saliva proteins, temperature changes from food and drinks, and the mechanical wear and tear from chewing and brushing your teeth. The mouth cavity's environment is complex and cannot be replicated in vitro. These variables, whether combined or separate, may influence the mechanical and physical characteristics of the materials, hence impacting the longevity of the prostheses (12 , 30).

## Conclusions

This study's findings indicated that immersion in mouthwashes adversely impacted the surface roughness of the 3D-printed resin material. Nonetheless, the hardness exhibited no substantial alterations post-exposure. Moreover, the contact angle was seen to decrease, indicating enhanced surface wettability. The results, especially the persistent hardness and enhanced wettability, indicate that 3D-printed resin may be an appropriate material for frequent mouthwash users.

## Figures and Tables

**Table 1:** Table Mouthwashes utilized in this study.

Mouthwash	Active components	Manufacturer	Alcohol percentage(%v/v)
Lacalut Aktiv	Aluminum lacalut, chlorhexidine digluconate, propylbis (2-hydroxyethyl) ammonium difluoride (olaflur)fluoridgehalt/fluoride content: 225 ppm	Dr. Theiss Naturwaren GmbH, Germany	0% Alcohol
Kin Forte	Zinc lactate, allantoin, panthenol, cetylpyridinium chloride, chlorhexidine digluconate 0.05%	Laboratorios Kin S.A., Spain	0% Alcohol
Corsodyl	Chlorhexidine digluconate 0.2 %	Smithkline Beecham Consumer Brands, Brentford, UK	7.6% ethanol

1

**Table 2:** Table Descriptive statistics for the surface roughness test results.

Groups	Mean	Std. Deviation	Std. Error	95% Confidence Interval forMeanLower Bound	95% Confidence Interval forMeanUpper Bound	One-way ANOVA	
						F	P-Value
D.W.	.840	.2716	.0859	.646	1.034	6.389	.001
Corsodyl	.940	.2119	.0670	.788	1.092		
Kin	1.250	.2759	.0872	1.053	1.447		
Lacalut	1.260	.3062	.0968	1.041	1.479		

2

**Table 3:** Table Descriptive statistics of the surface hardness test results.

	Mean	Std. Deviation	Std. Error	95% Confidence Interval forMeanLower Bound	95% Confidence Interval forMeanUpper Bound	One-way ANOVA	
						F	P
D.W.	89.360	1.7551	.5550	88.104	90.616	.224	.879
Corsodyl	89.300	1.6118	.5097	88.147	90.453		
Kin	89.330	1.6607	.5252	88.142	90.518		
Lacalut	88.830	1.6767	.5302	87.631	90.029		

3

**Table 4:** Table Descriptive statistics of wettability test results.

	Mean	Std. Deviation	Std. Error	95% Confidence Interval forMeanLower Bound	95% Confidence Interval forMeanUpper Bound	One way ANOVA	
						F	P
D.W.	82.14	0.222	0.0702	81.981	82.2989	87.61	.000
Corsodyl	77.10	1.259	0.3980	76.1967	77.9973		
Kin	76.03	0.7766	0.2456	75.4695	76.5805		
Lacalut	74.33	1.709	0.5404	73.1054	75.5506		

4

## Data Availability

The datasets used during the current study are available from the corresponding author.
